# Silencing of miR-150-5p Ameliorates Diabetic Nephropathy by Targeting SIRT1/p53/AMPK Pathway

**DOI:** 10.3389/fphys.2021.624989

**Published:** 2021-04-09

**Authors:** Wenmin Dong, Huiqian Zhang, Cheng Zhao, Yun Luo, Ying Chen

**Affiliations:** ^1^Shanghai TCM-Integrated Hospital Affiliated to Shanghai University of Traditional Chinese Medicine, Shanghai, China; ^2^Shanghai University of Traditional Chinese Medicine, Shanghai, China; ^3^Shanghai Research Institute of TCM Literature, Shanghai, China

**Keywords:** diabetic nephropathy, microRNAs, SIRT1, p53, autophagy

## Abstract

Diabetic nephropathy (DN) is a common complication of diabetes and an important cause of end-stage renal disease. Increasing evidence suggests that microRNAs (miRNAs) regulate the development of DN. In a preliminary study, high levels of miR-150-5p were detected in the serum and urine of patients with DN. Consequently, we investigated the effect and mechanism of action of miR-150-5p in DN *in vitro* and *in vivo*. Our results showed that inhibition of miR-150-5p reversed high glucose-induced podocyte injury and Streptozocin (STZ)-induced diabetic nephropathy in mice. Further analysis revealed that miR-150-5p targeted the 3′ untranslated region (UTR) of sirtuin 1 (SIRT1), consequently decreasing SIRT1 levels in podocytes. Importantly, we found that the silencing of miR-150-5p promoted the interaction between SIRT1 and p53, causing the suppression of p53 acetylation in podocytes and kidney tissue. This resulted in the stimulation of AMP-activated protein kinase (AMPK)-dependent autophagy. In conclusion, our study demonstrated that the silencing of miR-150-5p played a reno-protective role in DN mice through targeting SIRT1.

## Introduction

Diabetes is characterized by alteration in glucose metabolism, involving complex pathogenic mechanisms, leading to multiple complications. Diabetic nephropathy is one of the most serious complications of diabetes, which can lead to various microvascular diseases, causing glomerular sclerosis, and end-stage renal disease (ESRD) (Gross et al., 2005; Dronavalli et al., 2008). DN is the main cause of chronic kidney disease. Up to 50% of diabetic patients develop DN and eventually ESRD 20 years after onset of diabetes (Packham et al., 2012). It is estimated that 40–45% of type 1 diabetes patients and 30% of type 2 diabetes patients have diabetic nephropathy (Oltean et al., 2017). Strict control of blood pressure, administration of angiotensin-converting enzyme inhibitors, and inhibition of the renin–angiotensin system can alleviate the symptoms of DN. However, there are no effective drugs to prevent and treat DN. Therefore, it is important to understand the pathogenesis of DN in order to identify new drug targets (de Zeeuw, 2011).

Recently, there has been increasing studies showing that microRNAs (miRNAs) play an important role in the posttranscriptional regulation of genes in organisms that are closely associated with growth and development as well as pathogenesis of several diseases (Kim, 2005). There have been several studies showing that miRNAs play an important role in the pathogenesis of diabetic nephropathy (Chung, 2015; Assmann et al., 2018). For instance, Francesca et al. found that miRNA-27b-3p and miRNA-1228-3p in the urine were associated with the progression of renal fibrosis in diabetic kidney disease (DKD) (Conserva et al., 2019). In addition, inhibition of miR-21 ameliorated STZ-induced diabetic kidney disease in mice by targeting CDK6/CDC25 (Kolling et al., 2017). Xie et al. carried out microRNA sequencing on the exomes obtained from human urine and found that the miR-150-5p levels in DKD patients were significantly increased compared to the non-DKD patients (fold change, 3.477725) (Xie et al., 2017).

Based on previous studies, our aim was to investigate the role of miR-150-5p in high glucose-induced podocytes injury and STZ-induced diabetic kidneys mice.

## Materials and Methods

### Clinical Samples

A total of 60 patients with diabetes mellitus (DM) who had been admitted to the hospital between 2016 and 2018 were enrolled into this study. Serum and urine samples were collected within 24 h of onset of symptoms and immediately frozen in liquid nitrogen for further analysis. Patients were classified into two groups based on the degree of albuminuria: non-DN group (urinary albumin-to-creatinine ratio (UACR) < 2.5 mg/mmol and urinary albumin excretion rate (UAER) < 30 mg/24 h, *n* = 30) and DN group (UACR > 25 mg/mmol or UAER = 300–800 mg/24 h, *n* = 30). Ethical approval (permit number: 2015-092-1) for this work was given by the Independent Ethics Committee of Shanghai TCM-Integrated Hospital. Informed and written consent was obtained from all patients or their guardians according to the Ethics Committee guidelines.

### Animal

Male eNOS homozygous knockout (eNOS^−/−^) mice with C57BL/6J background were purchased from Caygen Biosciences Inc. (Guangzhou, China) and housed under specific pathogen-free conditions. This study was carried out in strict accordance with the Guide for the Care and Use of Laboratory Animals (Eighth Edition, 2011, published by The National Academies Press, 2101 Constitution Ave. NW, Washington, DC 20055, USA). The protocol was reviewed and approved by the Animal Care Committee of Shanghai TCM-Integrated Hospital (permit number PZSHUTCM201204008). Diabetes was induced in 8-week-old mice using intraperitoneal (I.P.) administration of STZ (Sigma, S0130, dissolved in 0.1 M citrate buffer, pH 4.5) at 50 mg/kg after 4–6 h of food deprivation each day for 5 consecutive days. Non-diabetic controls were injected with citrate buffer. Ten weeks after induction of diabetes, mice were given anti-miR-150-5p lentivirus, which was purchased from HanBio (Shanghai, China). The lentivirus cocktail was purified using filtration, and then, intravenous injections of 100 μl (1 × 10^5^ IU/μl) were administered weekly for 8 weeks. Mice that were administered 5% dimethyl sulfoxide (DMSO) served as controls for the lentivirus treatments. Mice were sacrificed 18 weeks after the onset of diabetes and the kidneys harvested for subsequent experiments. The surgery were performed under sodium pentobarbital anesthesia, and all efforts were made to minimize suffering.

### Urine Albumin Assessment

Urine albumin was detected using an ELISA kit (Nanjing Jiancheng Bioengineering Institute) according to the manufacturer's protocols.

### Cell Culture

Conditionally immortalized mouse podocytes were obtained from the Cell Bank at the Chinese Academy of Sciences (Shanghai, China) and cultured in Dulbecco's modified Eagle's medium (DMEM) containing 10% fetal calf serum.

### Statistical Analysis

All data were expressed as the mean ± standard error of the mean (SEM). Significant differences in mean were evaluated using one-way ANOVA in various groups accompanied by least significant difference (LSD) *post hoc* tests for mean separation. Two groups analysis was performed *t* test (two tailed). The significance level was set at *P* < 0.05.

Detailed information on materials and methods is shown in Supplementary Methods.

## Results

### Silencing of miR-150-5p Inhibits High Glucose-Induced Podocyte Injury

**Figure 1:**
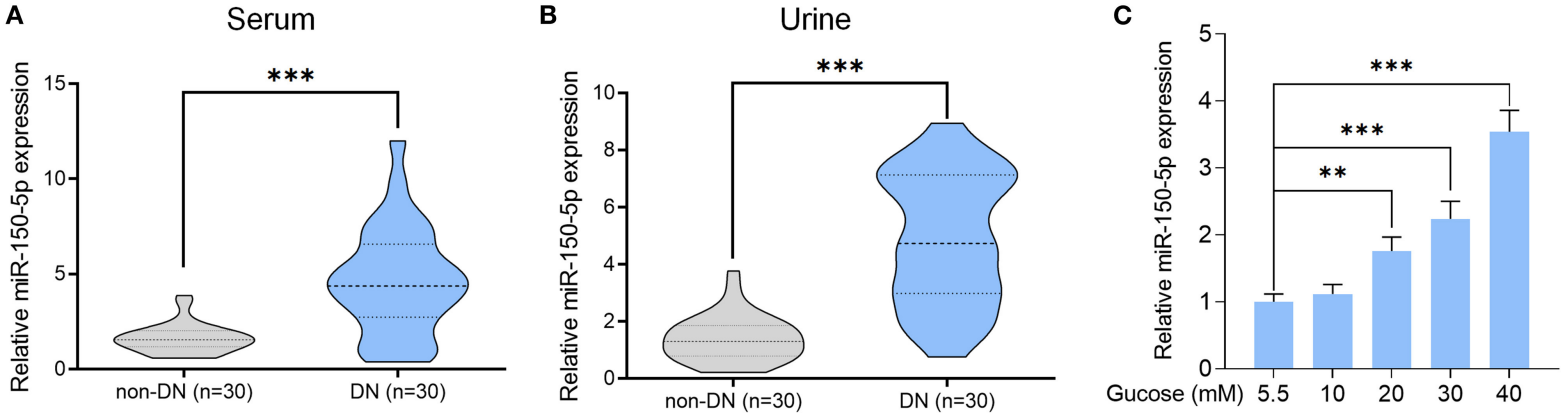
High glucose increased the level of miR-150-5p in the serum and urine of patients with diabetic nephropathy as well as in podocytes. **(A,B)** The level of miR-150-5p in the serum and urine of diabetic nephropathy (DN) patients was determined using quantitative real-time PCR (qRT-PCR). **(C)** The level of miR-150-5p in podocytes cultured in different concentrations of glucose was determined using qRT-PCR (*n* = 3). Data are expressed as mean ± SEM (**p < 0.01, ***p < 0.001).

Previous studies have shown abnormal levels of miR-150-5p in the urine samples of DN patients (Xie et al., 2017). In our study, we enrolled 60 DM patients that had been admitted in the hospital between 2016 and 2018 and divide these patients into DN (*n* = 30) and non-DN (*n* = 30) groups according to UACR and UAER (UACR > 25 mg/mmol or UAER = 300–800 mg/24 h for DN group and UACR < 2.5 mg and UAER < 30 mg/24 h for non-DN group). The clinical characteristics of the patients can be found in Table 1. To determine if there was any difference in the expression of miR-150-5p between DN and non-DN patients, we used qRT-PCR to evaluate the expression levels of miR-150-5p in the urine and serum samples of the patients. The results shown in Figures 1A,B revealed that there was a significant increase in the levels of miR-150-5p in the urine and serum samples of the DN patients.

**Table 1:** Baseline characteristics of patients with DM (*n* = 30).

**Characteristics**	**Non-DN (*n* = 30)**	**DN (*n* = 30)**	***p***
Age, mean ± SD, years	56.3 ± 18.2	65.9 ± 19.1	>0.05
Gender, n			>0.05
Men	17	15	>0.05
Women	13	15	>0.05
Smoking, *n*	13	11	>0.05
Hypertension, *n*	16	17	>0.05
Cardiovascular disease, *n*	5	4	>0.05
BMI, kg/m^2^	28.5 ± 6.5	29.1 ± 7.3	>0.05
UACR, mg/mmol	0.68 ± 0.21	49.3 ± 15.2	<0.05
UAER, mg/24 h	12.5 ± 9.3	527.3 ± 162.3	<0.05

Diabetic nephropathy is characterized by the loss of or damage to podocytes, which may be due to high glucose (HG) levels that are known to cause apoptosis of podocytes (Susztak et al., 2006). To investigate the role of miR-150-5p in podocytes, we cultured the podocytes in different concentrations of glucose for 48 h. As can be seen in Figure 1C, high levels of glucose induced the expression of miR-150-5p in a dose-dependent manner.

**Figure 2:**
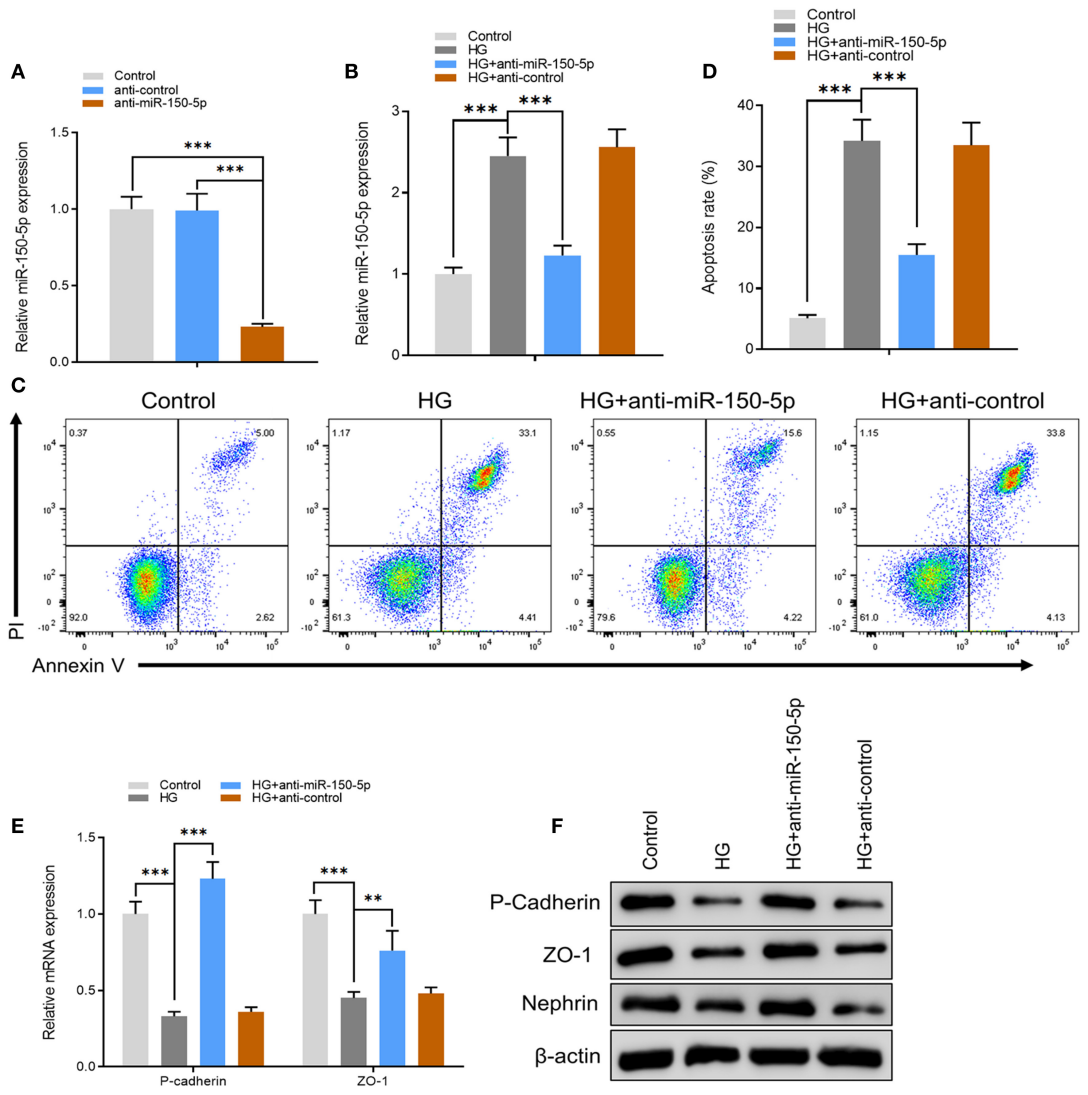
Silencing of miR-150-5p inhibited high glucose-induced podocytes injury. **(A)** The levels of miR-150-5p in podocytes were determined using quantitative real-time PCR (qRT-PCR) after transfection with anti-miR-150-5p for 48 h. **(B)** The levels of miR-150-5p in podocytes were determined using qRT-PCR after transfection with anti-miR-150-5p and culture in high glucose for 48 h. **(C,D)** Quantification and analysis of apoptosis rates using flow cytometry in podocytes after transfection with anti-miR-150-5p and culture in high glucose for 48 h. **(E,F)** The levels of P-cadherin and zonula occludens-1 (ZO-1) in podocytes were determined using qRT-PCR and Western blot after transfection with anti-miR-150-5p and culture in high glucose for 48 h. Data are expressed as mean ± SEM (*n* = 3; **p < 0.01, ***p < 0.001).

Next, we constructed an miR-150-5p knockdown vector (anti-miR-150-5p) and used it to transfect podocytes for 48 h. A comparison of miR-150-5p levels between transfected and non-transfected cells using quantitative real-time PCR (qRT-PCR) verified the efficacy of miR-150-5p knockdown (Figure 2A). The cells were then cultured in HG (30 mM) and transfected with anti-miR-150-5p vector. Results of qRT-PCR analysis showed that HG increased the expression of miR-150-5p compared to the control group (5.5 mM glucose), while transfection with anti-miR-150-5p decreased the HG-induced expression of miR-150-5p (Figure 2B). Flow cytometric analysis demonstrated that downregulation of miR-150-5p ameliorated high glucose-induced cell apoptosis in podocytes (Figures 2C,D). In diabetic nephropathy, podocytes undergo phenotypic switching. Podocytes can differentiate from epithelial cells to mesenchymal cells, which in turn causes the podocytes to lose their epithelial specificity and function (Thomas and Paul, 1996; Reidy and Susztak, 2009). The occurrence of epithelial–mesenchymal transition (EMT) in podocytes can be demonstrated by the negative regulation of the expression of biomarkers such as zonula occludens-1 (ZO-1), P-cadherin, and nephrin (Ying and Wu, 2017). Therefore, we evaluated the mRNA and protein levels of ZO-1, P-cadherin, and nephrin in the podocytes. As shown in Figures 2E,F, there was a significant decrease in the mRNA and protein levels of ZO-1, P-cadherin, and nephrin in the podocytes cultured in HG compared to the control group, which indicated that HG accelerated the EMT process in podocytes. As we had anticipated, anti-miR-150-5p reversed this HG-induced suppression, indicating that silencing of miR-150-5p ameliorates HG-induced function loss in podocytes.

### miR-150-5p Binds Directly to the 3′-UTR of SIRT1 and Inhibits Its Expression

**Figure 3:**
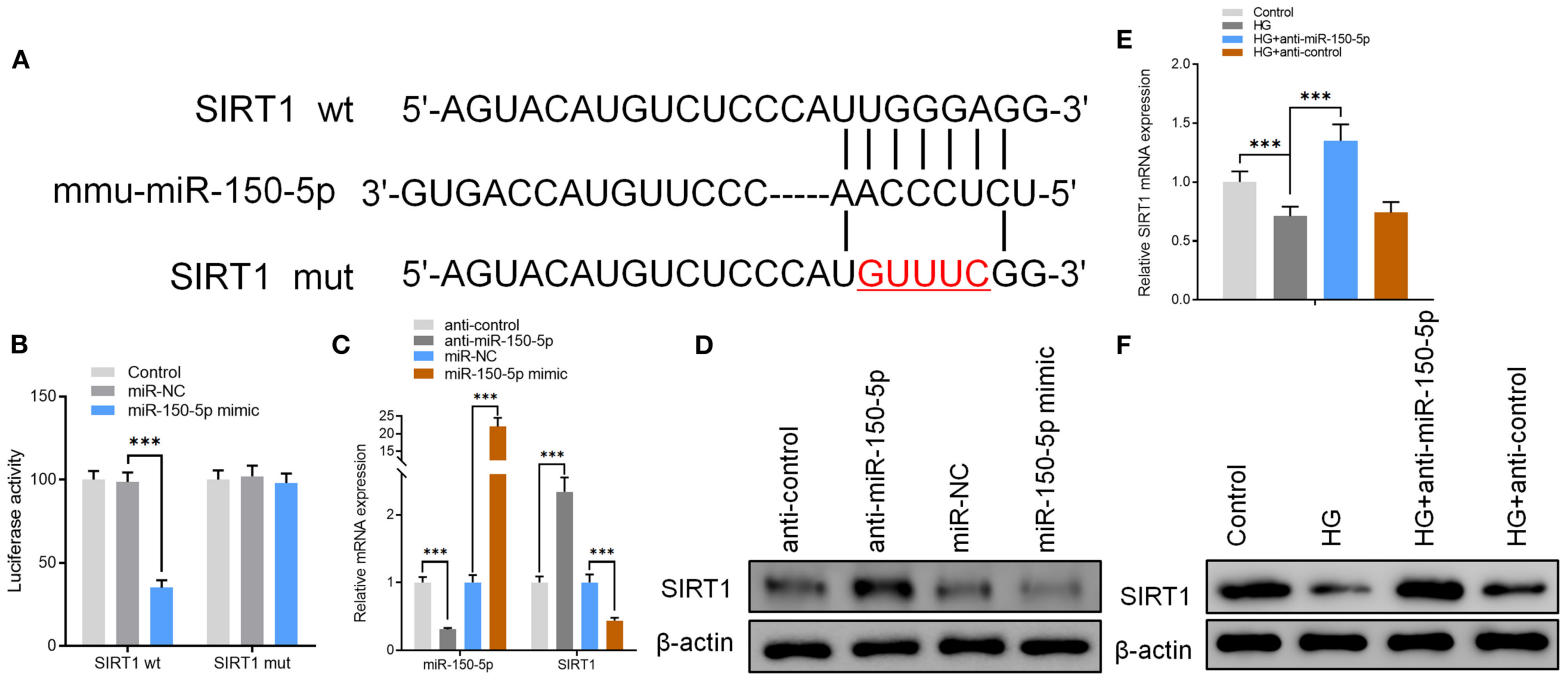
miR-150-5p bound directly to the 3′ untranslated region (UTR) of sirtuin 1 (SIRT1) and suppressed SIRT1 expression. **(A)** The predicted binding sites of miR-150-5p in the 3′ UTR of SIRT1. The mutated version of the SIRT1 3′ UTR is also shown. **(B)** The relative luciferase activity was determined in podocytes 48 h after transfection with the miR-150-5p mimic/control or the 3′ UTR of SIRT1 wt/mut constructs. **(C)** The levels of miR-150-5p and SIRT1 in podocytes were determined using qRT-PCR after transfection with anti-miR-150-5p or miR-150-5p mimic for 48 h. **(D)** The protein levels of SIRT1 in podocytes were determined using Western blot after transfection with anti-miR-150-5p or miR-150-5p mimic for 48 h. **(E)** The levels of SIRT1 in podocytes were determined using qRT-PCR and Western blot after transfection with anti-miR-150-5p and culture in high glucose for 48 h. **(F)** The protein levels of miR-150-5p and SIRT1 in podocytes were determined using Western blot after transfection with anti-miR-150-5p and culture in high glucose for 48 h. Data are expressed as mean ± SEM (*n* = 3; **p < 0.01, ***p < 0.001).

To further explore the mechanisms of action for miR-150-5p, miRNA target gene prediction software, miRanda and TargetScan, were used to predict the miRNA target genes. The prediction results showed that SIRT1, VEGFA, Notch3, and MMP14 have a potential binding site for miR-150-5p. However, only SIRT1 expression was altered after miR-150-5p knockdown in podocytes. To elucidate whether SIRT1 is a target of miR-150-5p, we constructed wild-type (wt) and mutant (mut) SIRT1 reporter plasmids. Co-expression of miR-150-5p and wild-type reporter plasmids significantly reduced the luciferase activity, while co-expression of miR-150-5p and mutated SIRT1 reporter significantly affected the luciferase activity in podocytes. These results showed that miR-150-5p directly targets SIRT1 (Figures 3A,B). Thereafter, the results of qRT-PCR and Western blot analysis revealed that silencing of miR-150-5p promoted the mRNA and protein levels of SIRT1, while on the other hand, overexpression of miR-150-5p (miR-150-5p mimic) suppressed the levels of SIRT1 (Figures 3C,D). In addition, there was a significant decrease in the mRNA and protein levels of SIRT1 when the podocytes were cultured in HG compared to the control, whereas the SIRT1 levels increased in the podocytes transfected with anti-miR-150-5p (Figures 3E,F).

### SIRT1 Mediates the Acetylation of p53 and Promotes Autophagy in Podocytes

**Figure 4:**
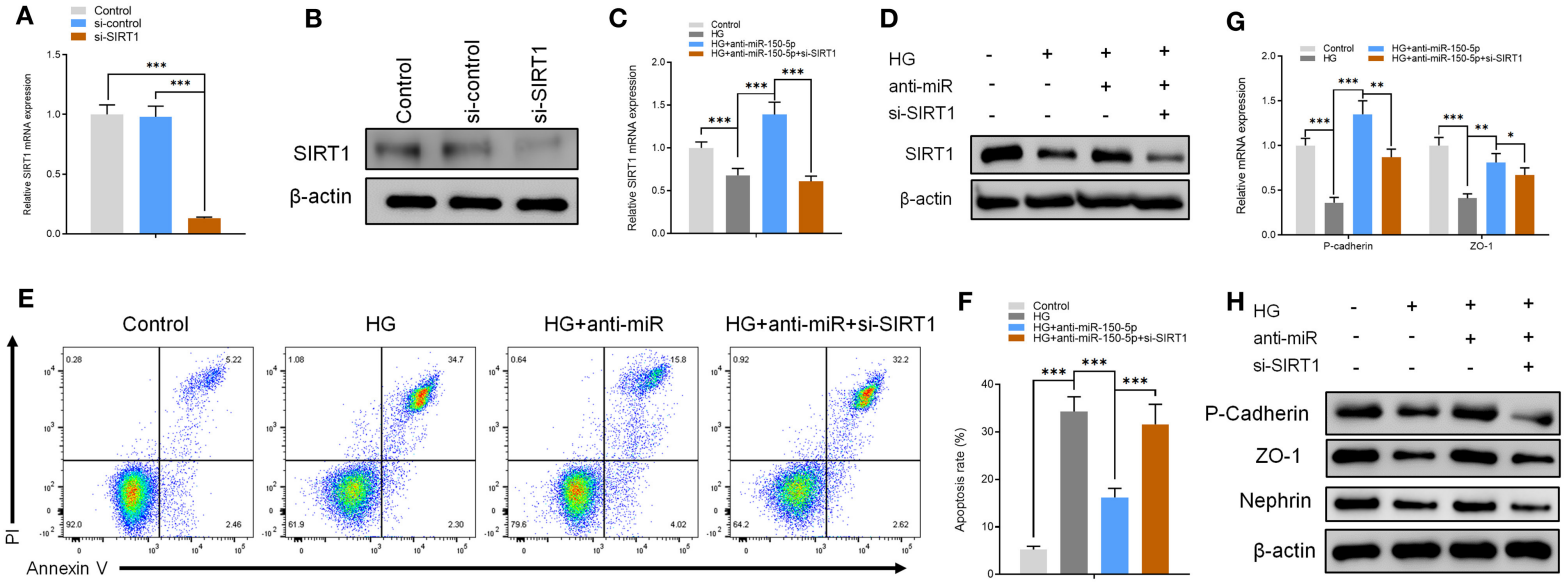
The effect of anti-miR-150-5p is mediated by sirtuin 1 (SIRT1) knockdown. **(A,B)** The levels of SIRT1 in podocytes were determined using qRT-PCR and Western blot after transfection with si-SIRT1 for 48 h. **(C,D)** The levels of SIRT1 in podocytes were determined using qRT-PCR and Western Blot after transfection with si-SIRT1 and culture in high glucose for 48 h. **(E,F)** Quantification and analysis of apoptosis rates using flow cytometry in podocytes after transfection with si-SIRT1 and culture in high glucose for 48 h. **(G,H)** The levels of P-cadherin and zonula occludens-1 (ZO-1) in podocytes were determined using qRT-PCR and Western blot after transfection with si-SIRT1 and culture in high glucose for 48 h. Data are expressed as mean ± SEM (*n* = 3; *p < 0.05, **p < 0.01, ***p < 0.001).

The functions of miR-150-5p were explored further through the knockdown of SIRT1 in podocytes. First, the podocytes were transfected with small interfering RNA against SIRT1 (si-SIRT1) and negative control (si-control). The results of qRT-PCR and Western blot analysis showed a remarkable decrease in the mRNA and protein levels of SIRT1 in podocyte transfected and non-transfected with anti-miR-150-5p in combination with si-SIRT1 (Figures 4A–D). Next, flow cytometry assays showed that the inhibitory effect of anti-miR-150-5p against HG-induced apoptosis in podocytes was reversed by si-SIRT1 (Figures 4E,F). Similarly, as shown in Figures 4G,H, si-SIRT1 suppressed the mRNA and protein levels of ZO-1, P-cadherin, and nephrin, which indicates that the inhibition of SIRT1 promotes the loss of podocyte function.

**Figure 5:**
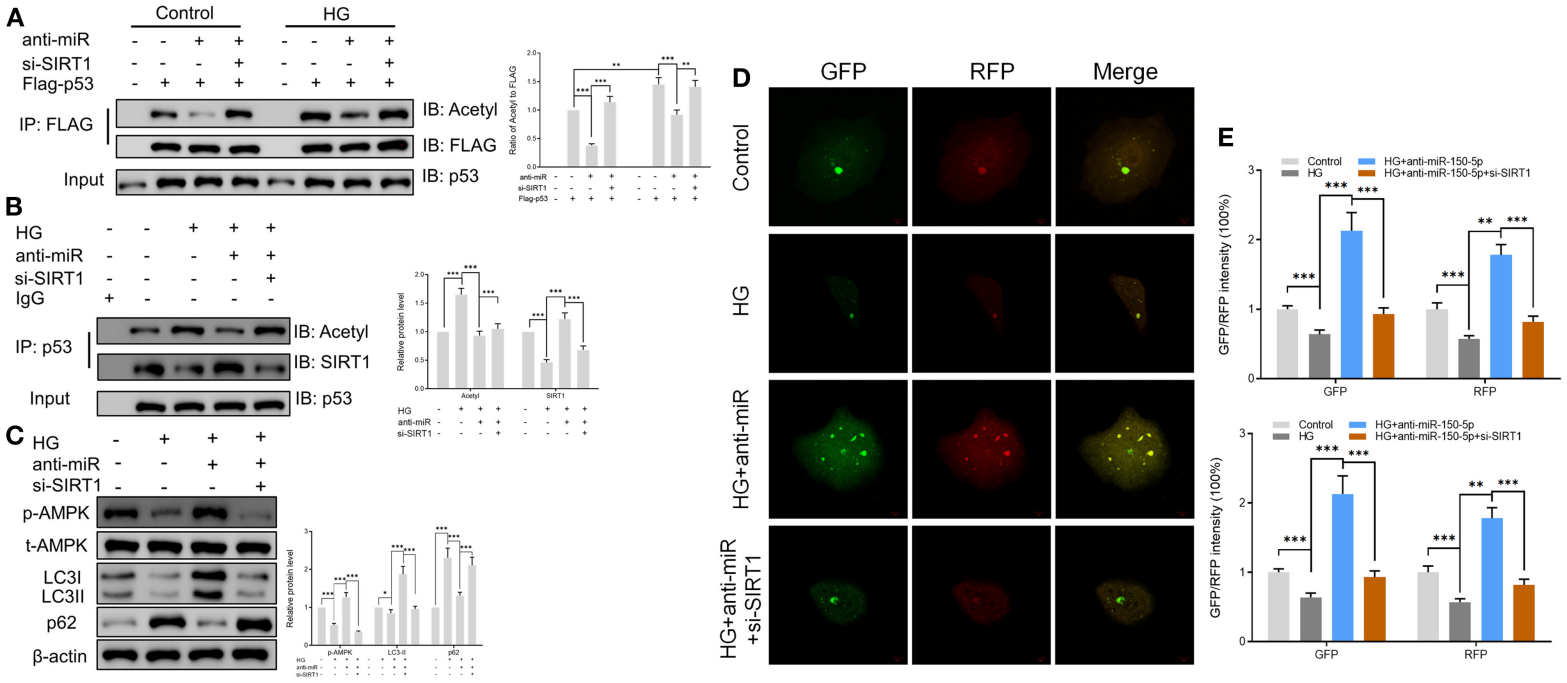
Sirtuin 1 (SIRT1) mediated the acetylation of p53 and promoted autophagy in podocytes. **(A)** p53 acetylation was determined in Flag IP, and total p53 was determined on total extracts as input in podocytes cultured in high glucose for 48 h after transfection with si-SIRT1 or anti-miR-150-5p and expressing Flag-p53. **(B)** Acetylation and SIRT1 were determined in p53 IP, and total p53 was determined on total extracts as input in podocytes cultured in high glucose for 48 h after transfection with si-SIRT1 or anti-miR-150-5p. **(C)** The levels of p-AMPK, LC-3, and p62 in podocytes were determined using Western blot after transfection with si-SIRT1 and culture in high glucose for 48 h. **(D,E)** Typical images of immunofluorescence staining of mRFP-GFP-LC3 in podocytes cells after transfection with si-SIRT1 and culture in high glucose for 48 h. Typical profiles of autophagosomes (RFP + GFP + dots) and autolysosomes (RFP + GFP-dots) per cell section tested by confocal microscopy are shown and quantified. Data are expressed as mean ± SEM (*n* = 3; *p < 0.05, **p < 0.01, ***p < 0.001).

SIRT1 is one of the most common histone deacetylases (Jesko et al., 2017). Several lines of evidence suggest that SIRT1 deacetylates p53 and promotes cell autophagy (De et al., 2018; Zhao et al., 2020); however, its role in podocytes remains unknown. In this study, we found that culturing of podocytes in high glucose media led to the acetylation of p53. However, anti-miR-150-5p decreased the HG-induced p53 acetylation, an effect that was reversed by si-SIRT1 (Figure 5A). Next, the extracts of podocytes were subjected to immunoprecipitation (IP)/Western blot assays with anti-SIRT1 as probes in p53-precipitated samples. As shown in Figure 5B, HG suppressed the interaction between SIRT1 and p53, whereas anti-miR-150-5p remarkably restored the interaction between them. Since previous studies have reported that p53 regulates the phosphorylation of AMPK and autophagy (Drakos et al., 2009; Jing et al., 2011), we investigated the involvement of AMPK in podocytes. Western blot analysis revealed that the silencing of miR-150-5p enhanced the phosphorylation of AMPK, thereby increasing the levels of LC3-II and decreasing the levels of p62, suggesting that anti-miR-150-5p promotes autophagy in podocytes (Figure 5C). Furthermore, the use of the autophagy biosensor, mRFP-GFP-LC3, revealed that HG suppressed the formation of autolysosomes (RFP) and autophagosomes (RFP) in podocytes, an effect that was inhibited by transfecting the podocytes with anti-miR-150-5p (Figures 5D,E).

### Silencing of miR-150-5p Ameliorates Kidney Injury in Type 1 Diabetic Mice

**Figure 6:**
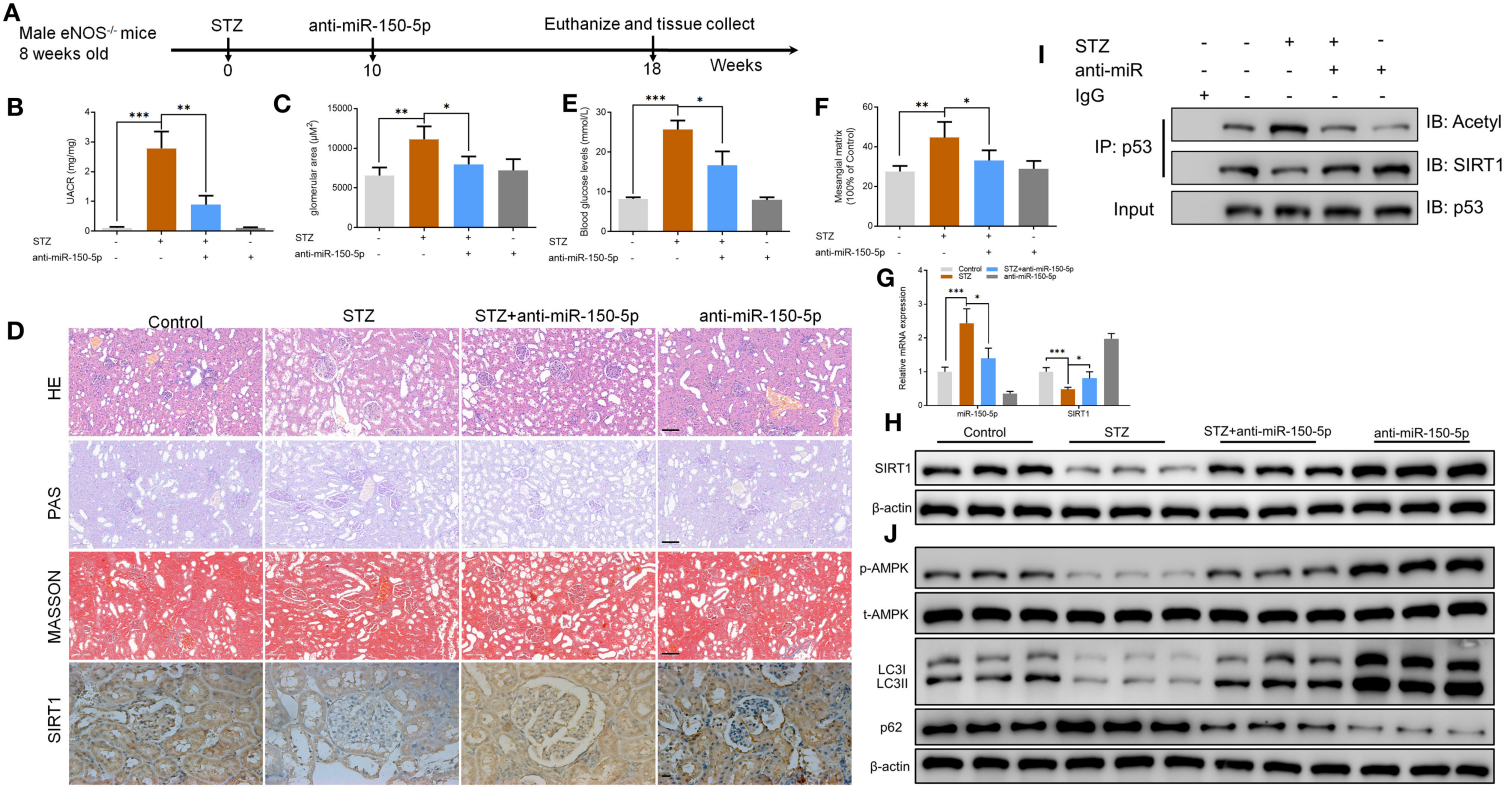
Silencing of miR-150-5p ameliorated kidney injury in type 1 diabetic mice. **(A)** eNOS^−/−^ mice were injected (i.p.) with STZ 50 mg/kg after 6 h of food deprivation each day for 5 consecutive days to induce diabetes. After 10 weeks, mice were given weekly intravenous injections of anti-miR-150-5p lentivirus for 8 weeks and then euthanized. **(B)** The level of urinary albumin-to-creatinine ratio (UACR) in mice. **(C)** The level of blood glucose in mice. **(D)** Representative images of hematoxylin–eosin (HE) (×200), periodic acid–Schiff (PAS) (×200), Masson-stained (×200), and immunohistochemistry of sirtuin 1 (SIRT1) (×400) in kidneys. Scale bar = 100 μm. **(E,F)** Quantification of the glomerular area and mesangial area fraction in mice kidney tissues. **(G)** The levels of miR-150-5p and SIRT1 in podocytes were determined using qRT-PCR in mice kidney tissues. **(H)** The protein levels of SIRT1 in podocytes were determined using Western blot in mice kidney tissues. **(I)** Acetylation and SIRT1 was determined in p53 IP, and total p53 was determined on total extracts as input in mice kidney tissues. **(J)** The levels of p-AMPK, LC-3, and p62 in podocytes were determined by Western blot in mice kidney tissues. Data are expressed as mean ± SEM (*n* = 6; *p < 0.05, **p < 0.01, ***p < 0.001).

To explore the function of miR-150-5p in diabetic nephropathy *in vivo*, we developed a type 1 diabetic model. In this model, eNOS^−/−^ mice were injected (i.p.) with STZ 50 mg/kg after 6 h of food deprivation each day for 5 consecutive days to induce diabetes. After 10 weeks, the mice received weekly intravenous injections of anti-miR-150-5p lentivirus for 8 weeks, after which the mice were euthanized (Figure 6A). UACR is a biomarker for diabetic nephropathy(Williams, 2005), and the results shown in Figures 6B,C reveal that there was a significant decrease in UACR and blood glucose in the mice after administration of anti-miR-150-5p. There were histomorphometry changes detected using H&E, PAS, and Masson staining in the renal tissue of DN mice (Figure 6D). These include the enlargement of the glomerular and mesangial matrix area in the mice administered with STZ, an effect that was reversed by transfection with anti-miR-150-5p (Figures 6E,F). Next, qRT-PCR results showed that the levels of miR-150-5p increased while the levels of SIRT1 decreased in the STZ group. The administration of anti-miR-150-5p restored the levels of miR-150-5p and SIRT1 to the control levels in mice kidney tissue. Similarly, the protein levels of SIRT1 were consistent with the mRNA levels in mice kidney tissue (Figures 6D,G,H). Further, the acetylation of p53 was determined using IP and Western blot. As shown in Figure 6I, STZ promoted the acetylation of p53 and inhibited the interaction between SIRT1 and p53, whereas anti-miR-150-5p significantly restored the interaction of SIRT1 and p53 in mice kidney tissue, which was consistent with the *in vitro* results. Next, Western blot results demonstrated that anti-miR-150-5p enhanced the phosphorylation of AMPK, which was suppressed by STZ treatment (Figure 6J).

## Discussion

Studies have shown that miRNAs are important small non-coding RNAs that mainly regulate the expression of target genes at the posttranscriptional level under physiological or pathological conditions (Kim et al., 2018). Recently, in-depth studies have identified additional miRNAs that have been implicated in the occurrence of diabetic nephropathy (Mafi et al., 2018; Yang et al., 2018b; Martinez and Peplow, 2019). Therefore, there is a need for further studies to determine the role of miRNAs in the regulation of diabetic nephropathy with the hope of discovering new therapies for diabetic nephropathy. Previous studies revealed that miR-150-5p plays various roles in the pathophysiology of diabetes. For instance, Che et al. (2020) found that the suppression of miR-150-5p ameliorated high glucose-induced myocarditis by targeting the SMAD7 pathway. In addition, miR-150-5p is also associated with β-cell injury caused by diabetes (Roat et al., 2019). Our study demonstrated that anti-miR-150-5p exerts reno-protective effects by targeting SIRT1 and restoring autophagy.

In a study on the relationship between SIRT1 and kidney disease, the expression levels of SIRT1 in the kidney tissues of diabetic rats were found to be significantly downregulated (Huang et al., 2019b). Shao et al. found that serum SIRT1 expression in diabetic patients decreased and its expression gradually decreased with the aggravation of proteinuria (Shao et al., 2017). Peter et al. constructed SIRT1 knockdown mice that showed severe albuminuria and mitochondrial dysfunction after Adriamycin-induced nephropathy, compared with wild-type mice (Chuang et al., 2014). Kazuhiro et al. found SIRT1 in proximal tubules protects against albuminuria in diabetes and influences podocyte function (Hasegawa et al., 2013). Previous research has demonstrated that SIRT1 deficiency in diabetic leads to hypoxia-inducible factor 1-alpha (HIF1α) activation, which leads to abnormal angiogenesis and fibrosis in the kidney (Takiyama and Haneda, 2014; Shao et al., 2016). Besides, SIRT1 prevents diabetic renal fibrosis by inhibiting the transforming growth factor beta 1 (TGF-β1)/Smad 2/3 pathway mediated EMT (Li et al., 2010; Yao et al., 2018). Zhang et al. (2018) reported that paeonol promoted the Nrf2/ARE pathway and inhibited oxidative stress through SIRT1 and alleviated diabetic renal injury in STZ-induced diabetic mice. What is more, silencing SIRT1 leads to the acetylation of FoxO3a, which aggravates the oxidative stress in HG-induced tubular epithelial cells (Wang et al., 2017). These results suggest that SIRT1 is closely related to oxidative stress injury and fibrosis in diabetic nephropathy. Through immunoprecipitation/Western blot assays, we demonstrated that SIRT1 deacetylates p53 in podocytes and mouse kidney tissues. However, we did not identify the specific acetylation sites for SIRT1 on p53. Further research using techniques such as liquid chromatography tandem mass spectrometry (LC-MS/MS) analysis will be necessary to determine the acetylation peptide and study the specific mechanisms of SIRT1–p53 posttranslational modification. This will be important for the identification of accurate targets for DN therapy.

Philippe et al. found that the phosphorylation of AMPK in the renal glomeruli and tubules of patients with DM was significantly reduced, suggesting that AMPK inactivation is involved in the progression of DN (Cammisotto et al., 2008). AMPK not only activates downstream signals in a SIRT1-dependent manner, but it is also upregulated by increasing cellular NAD+ levels due to SIRT1 activity (Wang et al., 2018; Huang et al., 2019a). This could cause AMPK activation by restricting glucose uptake and increase the activity of SIRT1 by promoting the transcription of NAD+ biosynthetic enzyme nicotinamide phosphoribosyltransferase (NAMPT) (Ding et al., 2010). Hence, in our future studies, we will focus on the effect of AMPK in regulating SIRT1 activity and investigate the possibility of forming a positive feedback loop in the high glucose environment of podocytes. Autophagy is considered to play an important role in the pathogenesis of various diseases (Doherty and Baehrecke, 2018). Increasing evidence shows that autophagy can regulate many key functions of the kidney in the normal and diseased state (Su et al., 2019; Zhang et al., 2020). STZ-induced autophagy is inhibited in the proximal and early tubules of DM rats, which is associated with renal tubular hypertrophy. Autophagy inhibition was also observed in distal tubules and could be reversed by insulin administration or islet transplantation (Eirin et al., 2017; Yang et al., 2018a). Inhibition of autophagy in podocytes was observed in STZ-induced DM mice, showing accumulation of autophagic degradation substrate p62 (Wang and Choi, 2014). In addition, autophagy is also involved in the maintenance of podocyte function, as suggested by the high rates of autophagy in podocytes and the effect of depletion of autophagy-related proteins on glomerulopathy in mice (Hartleben et al., 2010). Autophagy is modulated by nutrient state, and it changes under diabetic conditions, potentially exacerbating organelle dysfunction and leading to diabetic nephropathy (Yamahara et al., 2013). These results reveal that high glucose-induced changes on autophagy play an important role in diabetes-associated podocyte injury. However, in diabetic kidney disease, other than autophagy, other mechanisms, such as antioxidant and anti-inflammation effects, have been suggested to offer renal protection (Sun et al., 2019; Wang et al., 2019). Therefore, there is a need to investigate the involvement of other mechanisms in regulating the renoprotective function of miR-150-5p.

In summary, our work revealed that silencing of miR-150-5p potentially ameliorates high glucose-induced podocytes injury and STZ-induced mice diabetic nephropathy by targeting SIRT1, deacetylating p53, and restoring autophagy (Supplementary Figure 1). This study gives new insights into the renoprotective mechanisms of miR-150-5p in patients with diabetic nephropathy.

## Data Availability Statement

The raw data supporting the conclusions of this article will be made available by the authors, without undue reservation.

## Ethics Statement

The studies involving human participants were reviewed and approved by Independent Ethics Committee of Shanghai TCM-Integrated Hospital. The patients/participants provided their written informed consent to participate in this study. The animal study was reviewed and approved by the Animal Care Committee of Shanghai TCM-Integrated Hospital.

## Author Contributions

WD, YL, and YC designed and performed the experiments. WD and HZ performed the experiments and wrote the manuscript. WD and CZ analyzed the data, and YL and YC conceived the experiments.

## Conflict of Interest

The authors declare that the research was conducted in the absence of any commercial or financial relationships that could be construed as a potential conflict of interest.

## Supplementary Material

Supplementary Figure 1 Schematic representation of proposed mechanism of miR-150-5p on diabetic nephropathy.
