# Supplementary material for: Silencing of miR-150-5p Ameliorates Diabetic Nephropathy by Targeting SIRT1/p53/AMPK Pathway
Source: Front Physiol. 2021 Apr 9;12:624989. doi: 10.3389/fphys.2021.624989 (PMC8064124; doi:10.3389/fphys.2021.624989)
Supplement: Supplementary file 1 [file Data_Sheet_1.DOCX]

Silencing of miR-150-5p ameliorates diabetic nephropathy by targeting SIRT1/p53/AMPK pathway

Wenmin Dong^1,2#^, Huiqian Zhang^1,3#^, Cheng Zhao^1^, Yun Luo^1*^, Ying Chen^1,2*^

*1**. Shanghai TCM-Integrated Hospital affiliated to Shanghai University of Traditional Chinese Medicine, Shanghai, 200082, China*

*2. Shanghai University of Traditional Chinese Medicine, Shanghai,201203, China*

*3. Shanghai Research Institute of TCM Literature, 200020, China*

^#^ These authors contribute equally to this work

*Correspondence：

Ying Chen

Email: [lcys7382@126.com](mailto:lcys7382@126.com), Tel:13524922483

Shanghai TCM-Integrated Hospital affiliated to Shanghai University of Traditional Chinese Medicine, Shanghai, 200082, China

Shanghai University of Traditional Chinese Medicine, Shanghai,201203, China

Yun Luo

Email: [luoyun0504@163.com](mailto:luoyun0504@163.com), Tel: 18321839610

Shanghai TCM-Integrated Hospital affiliated to Shanghai University of Traditional Chinese Medicine, Shanghai, 200082, China

**Methods**

**Cell Culture**

Conditionally immortalized mouse podocytes were obtained from the Cell Bank at the Chinese Academy of Sciences (Shanghai, China) and cultured in DMEM containing 10% fetal calf serum.

**Cell Transfection**

For miR-150-5p and SIRT1 knockdown. anti- miR-150-5p and si-SIRT1 vector were constructed by GenePharma (Shanghai, China), and podocytes were transfected with either si-HMOX-1 or si-Sirt1 at a final concentration 50 nM using Lipofectamine 2000 (Invitrogen, Carlsbad, CA, United States) according to the manufacturer’s protocol.

**Flow Cytometry Assay**

Logarithmically growing podocytes were seeded into culture flasks. The cells were dual stained with Annexin V-FITC and propidium iodide (PI) for 30 min at room temperature. The stained cells were immediately analyzed by flow cytometry (Becton Dickinson, Franklin Lakes, NJ, United States). Apoptotic cells were defined as Annexin V-FITC positive and PI negative.

**RNA Isolation and Quantitative Real-Time PCR**

RNA was isolated using the TRIzol reagent (Invitrogen) according to the manufacturer’s instructions in podocytes and mouse kidney samples and reverse transcribed using a miScript Reverse Transcription kit (Qiagen). QRT-PCR was performed using the SYBR Premium Ex Taq II kit (Takara, Dalian, China) in an ABI PRISM 7500 Sequence Detection System (Applied Biosystems). All reactions were performed in triplicate and the mean value was used to calculate expression levels after normalization to β-actin as an internal standard.

**Immunoprecipitation**

For immunoprecipitation studies, 5 μg of anti-p65 or Rabbit IgG-AC (Proteintech 10746-1-AP: Santa Cruz, Dallas, TX, United States, ab37415) was added to cell lysates and incubated overnight at 4°C, under constant rotation. Immune complexes were precipitated and washed in lysis buffer. Immunoprecipitated samples were subject to western blotting analysis by using anti-acetylation antibody (Santa Cruz, Dallas, TX, United States, ab51997) and anti-Flag or anti-p65 antibody.

**Protein Extraction and Western Blot Analysis**

Podocytes were lysed using RIPA buffer, and protein concentration was determined using the BCA protein assay kit. Approximately 30 μg of protein from each sample was separated using a 10% SDS-polyacrylamide gel and transferred to PVDF membranes. Membranes were blocked with 5% skim milk in TBST and incubated with primary antibodies overnight at 4°C. Membranes were then incubated with the corresponding secondary antibodies for 1 h at room temperature and washed in TBST. Proteins were detected using specific antibodies: SIRT1 (#8469), Flag (#14793), p53 (2524), p-AMPK (#50081), AMPK (#5831), LC3B (#3868), p62 (#39749), β-Actin (#3700).

**Autophagic Flux Analysis**

Cells transfected with mRFP-GFP-LC3 were fixed with 4% paraformaldehyde and stained with 10 mM Hoechst 33342. Cell images were obtained using the Operetta High Content Imaging System (Perkin-Elmer) and analyzed using Harmony Analysis Software (Perkin-Elmer). Cells were analyzed using green (GFP) or red (mRFP) fluorescence. Autophagosomes were stained yellow puncta and autolysosomes stained red puncta in merged images. Autophagic flux was determined by the increased

percentage of red puncta in merged images.

**Kidney histology**

Kidneys were removed and fixed with 4% paraformaldehyde 16 h at 4 °C. The 4-μm sections were cut from paraffin-embedded kidney tissues. Sections were stained with PAS for histology analysis. Assessment of the mesangial and glomerular cross-sectional areas was performed by pixel counts on the kidney section in a blinded fashion, under × 400 magnification (Olympus, Tokyo, Japan) as previously described32,33. In brief, digitized images were scanned and profile areas were traced using ImageJ. The mean glomerular tuft volume was determined from the mean glomerular cross-sectional area by light microscopy. The glomerular cross-sectional area was calculated based on the average area of 30 glomeruli in each group as previously described [[1](#_ENREF_1)].

**Reference**

[1] Zhong, Y.; Lee, K.; Deng, Y.; Ma, Y.; Chen, Y., et al. Arctigenin attenuates diabetic kidney disease through the activation of PP2A in podocytes, Nature communications.10 (2019) 4523, https://doi.org/10.1038/s41467-019-12433-w.
