# Supplementary figures and images for: Silencing of miR-150-5p Ameliorates Diabetic Nephropathy by Targeting SIRT1/p53/AMPK Pathway
Source: Front Physiol. 2021 Apr 9;12:624989. doi: 10.3389/fphys.2021.624989 (PMC8064124; doi:10.3389/fphys.2021.624989)

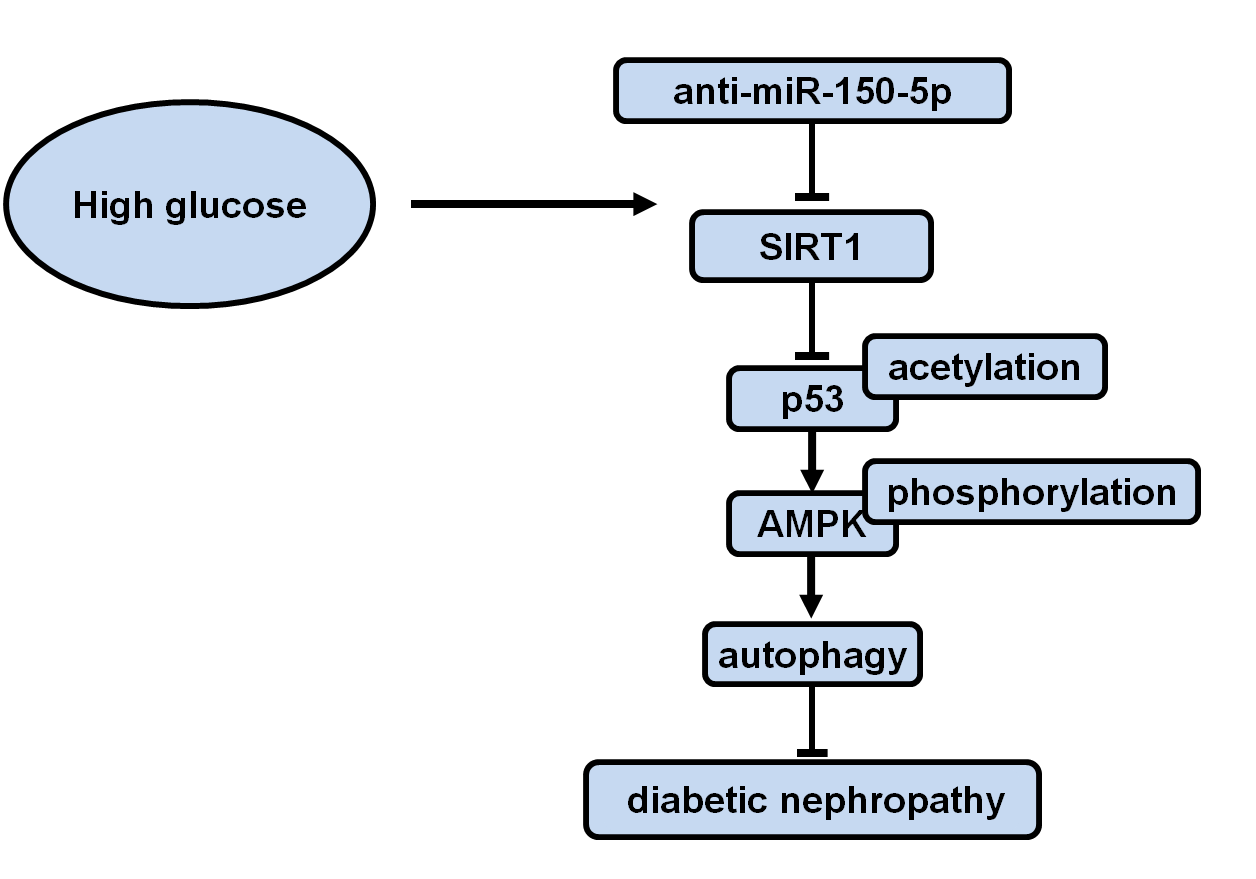

Supplement: Supplementary Figure 1 — Schematic representation of proposed mechanism of miR-150-5p on diabetic nephropathy. [file Image_1.TIF]
